# Ferroptotic MSCs protect mice against sepsis via promoting macrophage efferocytosis

**DOI:** 10.1038/s41419-022-05264-z

**Published:** 2022-09-26

**Authors:** Yuchen Pan, Jingman Li, Jiali Wang, Qi Jiang, Jingjing Yang, Huan Dou, Huaping Liang, Kuanyu Li, Yayi Hou

**Affiliations:** 1 State Key Laboratory of Pharmaceutical Biotechnology, Division of Immunology, Medical School, Nanjing University, 210093 Nanjing, China; 2 Jiangsu Key Laboratory of Molecular Medicine, 210093 Nanjing, China; 3 State Kay Laboratory of Trauma, Burns and Combined Injury, Research Institute of Surgery, Daping Hospital, The Army Medical University, 400042 Chongqing, China

**Keywords:** Immunology, Mesenchymal stem cells, Innate immune cells

## Abstract

The therapeutic effect of mesenchymal stem cells (MSCs) on sepsis has been well-known. However, a comprehensive understanding of the relationship between MSCs and macrophages remains elusive. Superparamagnetic iron oxide (SPIO) is one of the most commonly used tracers for MSCs. Our previous study has shown that SPIO enhanced the therapeutic effect of MSCs in a macrophage-dependent manner. However, the fate of SPIO-labeled MSCs (MSC^SPIO^) after infusion remains unknown and the direct interaction between MSC^SPIO^ and macrophages remains unclear. Mice were injected intravenously with MSC^SPIO^ at 2 h after *Escherichia coli* infection and sacrificed at different times to investigate their distribution and therapeutic effect. We found that MSC^SPIO^ homed to lungs rapidly after infusion and then trapped in livers for more than 10 days. Only a few MSC^SPIO^ homed to the spleen and there was no MSC^SPIO^ detectable in the brain, heart, kidney, colon, and uterus. MSC^SPIO^ tended to stay longer in injured organs compared with healthy organs and played a long-term protective role in sepsis. The mRNA expression profiles between MSCs and MSC^SPIO^ were rather different, genes related to lipid metabolism, inflammation, and oxidative stress were changed. The levels of ROS and lipid peroxide were elevated in MSC^SPIO^, which confirmed that SPIO-induced ferroptosis in MSC^SPIO^. Ferroptosis of MSC^SPIO^ induced by SPIO enhanced the efferocytosis of macrophages and thus enhanced the protective effect on septic mice, while the benefits were impaired after MSC^SPIO^ were treated with Ferrostatin-1 (Fer-1) or Liproxtatin-1 (Lip-1), the inhibitors of ferroptosis. SPIO-induced ferroptosis in MSCs contributes to better therapeutic effects in sepsis by enhancing the efferocytosis of macrophages. Our data showed the efficacy and advantage of MSC^SPIO^ as a therapeutic tool and the cell states exert different curative effects on sepsis.

## Introduction

Sepsis is defined as life-threatening organ dysfunction that is caused by a disordered host response to infection and has been a major socioeconomic burden worldwide [1, 2]. The effectiveness and safety of mesenchymal stem cells (MSCs) have been broadly investigated in animal models and clinical trials on sepsis (http://clinicaltrials.gov). With few infusion reactions and adverse reactions, MSCs are currently studied as a therapeutic strategy for sepsis [3] and COVID-19 patients [4, 5]. However, after intravenous injection, the majority of MSCs in healthy mice were present in the lungs within 5 min [6], yet the harsh environment in septic mice may cause the death of MSCs. The fate of MSCs and the mechanism of their action are largely unknown, and the impact of different cell states on sepsis remains controversial.

We noticed that MSCs administration reduced bacterial burden in septic mice [7, 8]. Since MSCs are unable to kill or clear bacteria, the benefit of MSCs treatment may largely be due to their immunomodulatory properties. Moreover, reports recently showed that most MSCs are fragmented and co-localized with monocytes-macrophages in the liver at 24 h post-infusion [9], suggesting that MSCs may be dying or have been dead when they interacted with macrophages to augment macrophage-mediated phagocytosis and bacterial killing [10]. Importantly, one critical process of macrophages is to clear the dying cells, also termed efferocytosis, which prevents inflammation by preventing cell necrosis [11]. Efferocytosis promotes tissue repair by inducing macrophage proliferation via nucleic acids [12]. In particular, macrophages that received the “find-me” or “eat-me” signals have to touch the dying cells to perform efferocytosis, thus the therapeutic effect of MSCs is largely dependent on their homing behavior [13]. In that case, the way to monitor MSCs in vivo is important for improving their therapeutic strategy.

Various tracers have been invented to investigate the fate of MSCs in vivo. Among them, superparamagnetic iron oxide (SPIO) is one of the most commonly used tracers for MSCs [14], and has been approved by FDA for clinical use [15, 16]. SPIO is generally considered safe, low toxicity, inert, metabolizable, and suitable for magnetic resonance imaging (MRI) in vivo [17]. However, SPIO labeling leads to iron deposition in MSCs, which is a typical characteristic of ferroptosis [18, 19]. Ferroptosis, which is proposed in 2012, is an iron-dependent new type of programmed cell death and is different from apoptosis, necrosis, and autophagy [20, 21]. These combined data raise the possibility that ferroptosis of MSCs induced by SPIO may induce the efferocytosis of macrophages and thus relieve sepsis-induced organ damage.

In our previous study, we found that SPIO treatment enhanced the effect of MSCs on educating macrophages polarized into the M2 subtype, and SPIO-treated MSCs had a stronger therapeutic effect on sepsis [22]. In our present study, we determined the spatiotemporal distribution of MSCs in healthy and septic mice. We found that the microenvironment significantly affected the fate of MSCs and the MSCs tend to be enriched in the damaged lungs and livers and played long-term protection. We verified that SPIO-induced ferroptosis of MSCs could promote the efferocytosis of macrophages, and reprogram macrophages to an anti-inflammatory phenotype, thereby protecting mice against sepsis. Our data suggest that the cross-talk between exogenous MSCs and host immune cells is crucial for cell therapy.

## Materials and methods

### Reagents

Prussian-blue kit (Solarbio, Beijing, China), ROS Assay kit (Beyotime, Shanghai, China), Annexin V-FITC/PI Apoptosis Detection Kit (Vazyme, Nanjing, China), CCK-8 kit, and Liperfluo (Dojindo, Shanghai, China) were used following instructions. All ELISA kits were purchased from Dakewe (BioLegend, California, USA). Ferrostatin-1 (HY-100579), Liproxtatin-1 (HY-12726) and RSL3 (HY-100218A) was purchased from MedChemExpress (MCE, Monmouth Junction, NJ, USA) and prepared in DMSO. M-CSF (Novoprotein, Shanghai, China) for BMDM was prepared in ddH_2_O. NIR-797 and Rhodamine B (Hualanchem, Shanghai, China) were prepared in DMSO. The antibodies used for flow cytometry (supplemental Table S1) and PCR Assay plates (Wcgene Biotech, Shanghai, China) were all purchased from fcmacs (Nanjing, China). SPIO nanoparticles (Ferumoxytol) were kindly provided by professor Ning Gu from Southeast University.

### Cells and culture conditions

Bone marrow-derived macrophages (BMDMs) were cultured in DMEM (Gibico, Grand iSland, NY, USA) with 10 ng/mL M-CSF, and MSCs were cultured in DMEM/F-12 (Gibico, Grand iSland, NY, USA), both containing 10% FBS (Gibico), 1% penicillin and streptomycin (100 μg/mL; Gibco BRL, USA), at 37 °C in a humidified atmosphere with 5% CO_2_.

### Bacterial strain

The clinical strains of *E*. *coli* were isolated from human clinical specimens and identified by the Medical Laboratory Center of Zhongda Hospital in Nanjing, Jiangsu, China. Bacterial strains were stored at –80 °C and prepared in LB Medium or LB-Agar medium before use.

### Mice

Female ICR mice at 6–8 weeks old were obtained from the Sino-British SIPPR/BK Lab. Animal Ltd (Shanghai, China). They were acclimatized for 5–7 days before molding. All procedures involving animals were in strict accordance with protocols approved by the Research Ethics Committee of Nanjing University. Mice were housed in specific pathogen-free conditions at the Nanjing University Animal Care Commission. Mice were grouped randomly. At the end of the experiment, mice were terminated humanely.

### Experimental sepsis

*E*. *coli* grown to mid-exponential phase were harvested, washed, and resuspended with normal saline. Mice were injected intraperitoneally (i.p.) with 0.2 mL bacterial suspension. Early removal criteria were described previously [23].

### Histology and tissue injury scoring

Lung and liver tissue sections were stained with hematoxylin and eosin (H&E) and observed under a light microscope. Morphological changes in lung and liver tissue were scored as described before [23].

### Synthesis of SPIO-RhB or SPIO-NIR

First, SPIO (20 mg) and Rhodamine B isothiocyanate (RhB; 0.5 mg; CAS: 36877-69-7) or NIR-797 (NIR; 0.5 mg; CAS: 152111-91-6) were added into a 10 mL flask with 5 mL dimethyl sulfoxide (DMSO; Sigma). The mixture was stirred overnight at room temperature and then diluted with 20 mL of deionized water. The diluted mixture was dialyzed against 2 L of ddH_2_O in a 3.5 kDa MWCO for two days to remove the unreacted RhB or NIR. The products were dried under reduced pressure to obtain fluorescence-SPIO (SPIO-RhB or BSNP-NIR).

### Enzyme-linked immunosorbent assay (ELISA)

TNF-α, IL-1β, and IL-6 were measured using ELISA kits according to the manufacturer’s protocol. Supernatants of cells were collected and stored at −80 °C until assayed.

### Immunofluorescence

For immunofluorescence analysis, cells were seeded on the plates pre-coated with cover slides. At the end of the experiment, 4% paraformaldehyde (PFA) was added to each pore for 10 min. Then cells were washed with ddH_2_O at least 3 times. The cover slides were then placed on the glass slide with an anti-fluorescence quenching agent. Fluorescence images were obtained using a confocal microscope (FV3000, Olympus, Japan / Stellaris, Leica, Germany).

### RNA extraction and quantitative real-time PCR

Total RNA was isolated using Trizol Reagent (Vazyme, Nanjing, China) according to the manufacturer’s instructions. Collected mRNA was reverse-transcribed to cDNA using HiScript ® II Q RT SuperMix kit (Vazyme). Real-time PCR assay was then performed using SYBR green dye (Invitrogen) on the StepOne sequence detection system (Applied Biosystems, Waltham, MA, USA). The relative abundance of genes was calculated by using the 2^−△△CT^ formula. The sequences of the qRT-PCR primers are provided in Supplemental Table S2. PCR Array assay was performed according to the manufacturer’s protocol (Wcgene Biotech, Shanghai, China) on the StepOne sequence detection system.

### Statistical analysis

All of the values presented on the graphs are given as means ± S.E.M. ANOVA and unpaired Student’s *t*-tests were used to analyze the statistical significance. All experiments were repeated at least three times. Statistical differences were defined as significant for *p < 0.05 and highly significant for **p < 0.01 and ***p < 0.001. GraphPad Prism5 Demo (GraphPad Software Inc., La Jolla, CA, USA) was used for statistical analysis.

## Results

### Characterization of MSCs and MSC^SPIO^

**Fig. 1:**
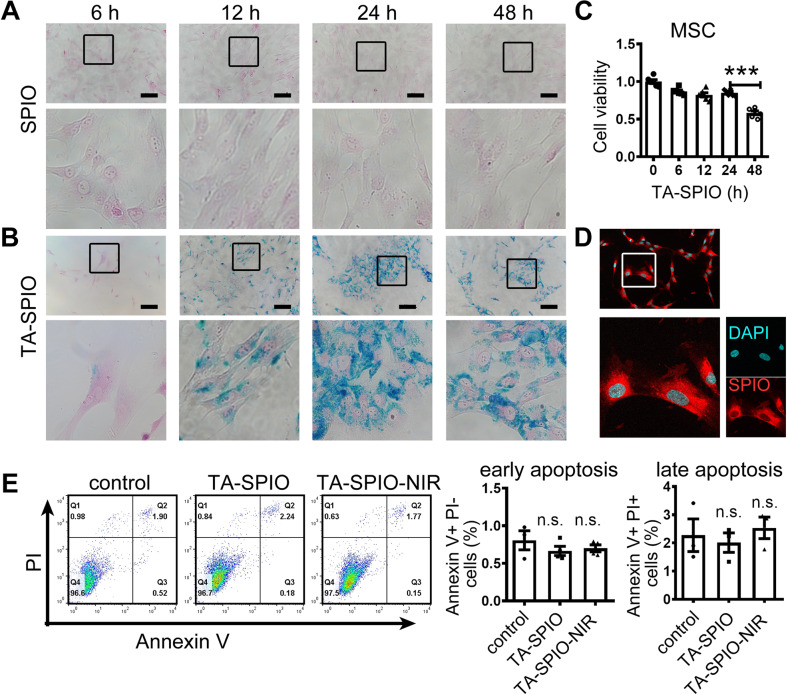
Characterization of MSCs and MSC^SPIO^. **A**, **B** Prussian-blue staining of MSCs treated with **A** SPIO alone, or **B** SPIO and transfection agent. **C** MSCs were treated with SPIO and transfection agent, cell viability was measured by CCK-8 assay. **D** MSCs were treated with SPIO-NIR and transfection agent for 24 h, the fluorescence intensity was measured by confocal microscope. **E** Cell apoptosis was determined by flow cytometry. Data are shown as mean ± SEM (*n* = 3). ***p < 0.001. TA transfection agent (Superfect, Qiagen).

When MSCs were treated with SPIO alone, their cellular iron was hardly detectable at Prussian-blue staining (Fig. 1A). When MSCs were incubated with SPIO and transfection agent, iron was detectable at 6 h and numerous iron-containing vesicles showed up after 24 h (Fig. 1B). CCK-8 assay showed that the cell viability of MSCs was slightly decreased within 24 h while it remarkably decreased at 48 h after treatment of SPIO and transfection agent (Fig. 1C). Therefore, MSCs were incubated with SPIO and transfection agent for 24 h as the process of SPIO-labeled MSCs (MSC^SPIO^) in subsequent experiments. Then, we coupled SPIO with NIR-797 fluorescein (SPIO-NIR) for better visualization. All MSCs labeled with SPIO-NIR (MSC^SPIO-NIR^) showed intense fluorescence (Fig. 1D and Supplemental Fig. S1A, B). Both SPIO and SPIO-NIR have little effect on MSCs apoptosis (Fig. 1E) and their surface markers (Supplemental Fig. S1C).

### MSC^SPIO^ can be recruited to impaired organs and reduce organ damage

First, for a study of labeling stability, both MSC^SPIO^ and MSC^SPIO-NIR^ were washed 3 times with PBS and cultured with fresh culture medium for another few days. The results of confocal microscope and Prussian-blue staining showed that the SPIO signal decayed as the cells proliferated, yet dim fluorescence and small blue dots exist 6 days after labeling (Supplementary Fig. S2A, B). Since MSCs would proliferate much lower in mice than in vitro, our results suggest that SPIO can track MSCs in vivo for at least a week.

**Fig. 2:**
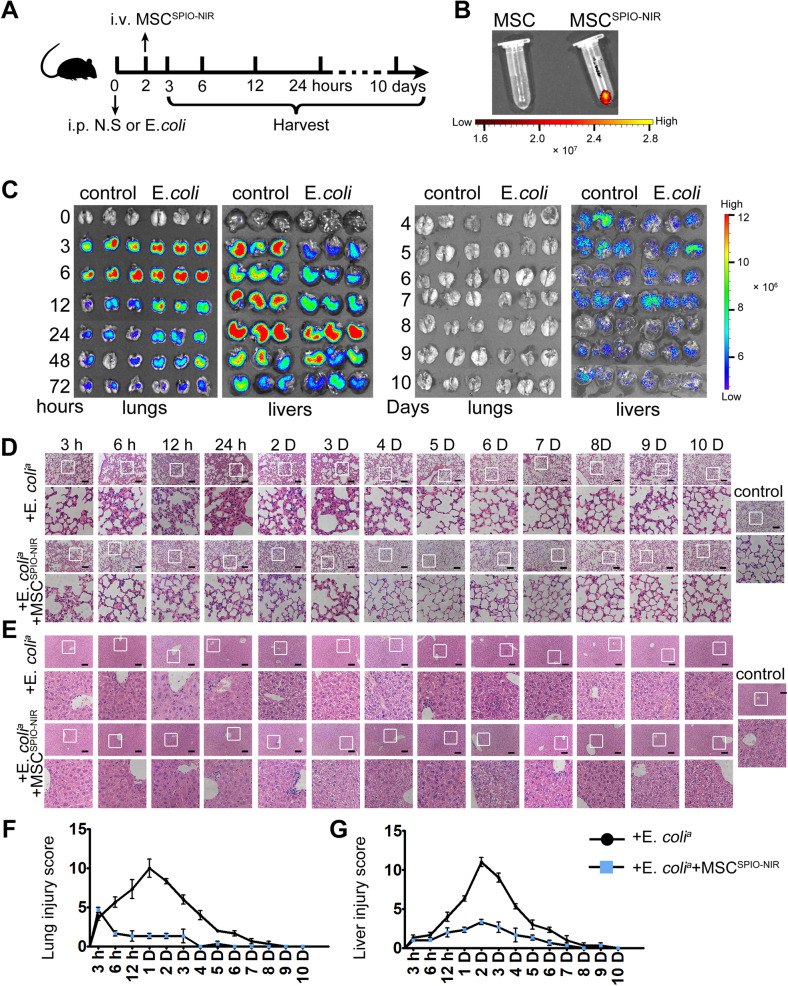
The spatiotemporal distribution and therapeutic effect of MSC^SPIO-NIR^. **A** Schematic of the MSC^SPIO-NIR^ tracing experiment setup (*n* = 3). **B**, **C** The ex vivo imaging of **B** EP tubes, **C** lungs, and livers were carried out on the In Vivo Imaging System. Representative H&E staining of **D** lung sections or **E** liver sections. Scale bars, 100 μm. Histological injury of **F** lungs and **G** livers were scored as described in Materials and Methods. Data are shown as mean ± SEM (*n* = 3).

Then SPIO were coupled with small-molecule fluorescein NIR-797 to better determine the spatiotemporal distribution of cells in vivo. MSC^SPIO-NIR^ were harvested and washed with PBS, and then prepared for injection. Mice were first injected intraperitoneally with normal saline or *E*. *coli* and injected intravenously with MSC^SPIO-NIR^ 2 h later. Then the mice were sacrificed to obtain organs at different times (Fig. 2A, B). Ex vivo imaging showed localization of MSC^SPIO-NIR^ mainly in the lungs and livers of mice (Fig. 2C), while the fluorescence was faint in the spleen and was hardly detectable in the brain, heart, kidney, colon, or uterus (Supplemental Fig. S3). Shortly after MSC^SPIO-NIR^ injection, the fluorescence intensity in the lungs of septic mice was stronger than that of healthy mice (Fig. 2B). Correspondingly, the fluorescence intensity in the livers of septic mice was lower than that of healthy mice (Fig. 2C). Lung fluorescence decreased from 6 h and was not detectable after 4 days, while liver fluorescence decreased from 1 day and maintain detectable after MSC^SPIO-NIR^ injection (Fig. 2C).

By analyzing the pathological slides of the lungs, we found that the inflammatory cells infiltrated rapidly after *E*. *coli* injection, and the alveolar walls incrassated and became hyperemic within 6 h after infection. Severe lung damage could last up to 3 days and then recover gradually without therapy (Fig. 2D). MSC^SPIO-NIR^ treatment remarkably decreased incrassation, hyperemia, and exudates in the alveolar walls during the early stage of sepsis. The lung sections of mice in the treatment group were closer to those in the control group at any time point (Fig. 2D, F). Moreover, the inflammatory cell infiltrated the livers till 12 h and severe vacuolization occurred 1 days after the infection. Liver damage mainly occurred in the late stages of sepsis and MSC^SPIO-NIR^ treatment remarkably decreased sinusoidal congestion and vacuolization in the liver (Fig. 2E, G).

Collectively, our results indicate that MSC^SPIO^ mainly aggregate to the damaged site of septic mice and thus inducing long-term protection against sepsis.

### SPIO labeling induces ferroptosis of MSCs

**Fig. 3:**
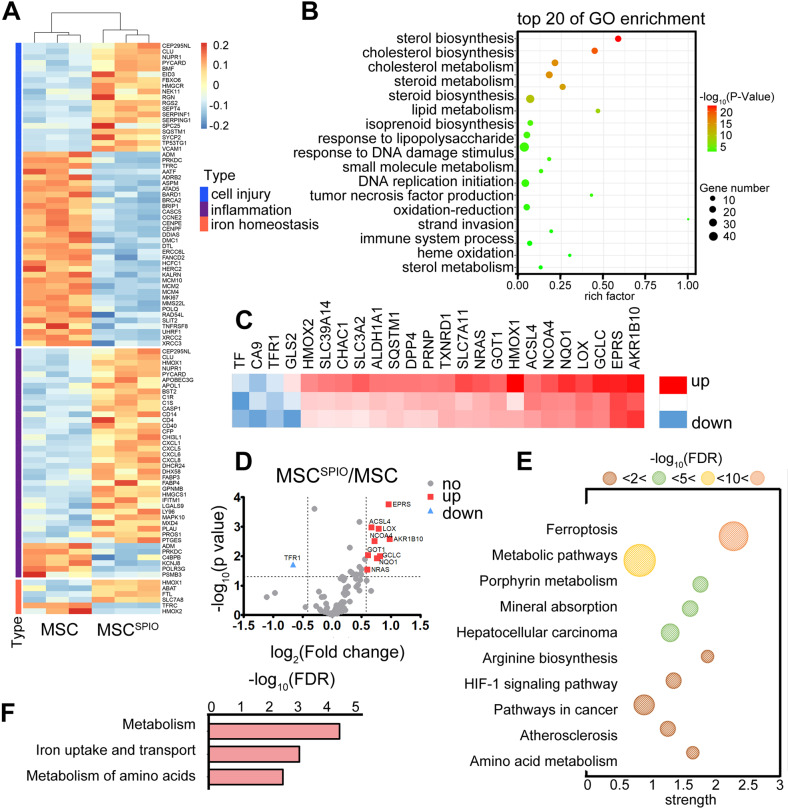
Expression pattern of genes in MSCs and MSC^SPIO^. **A** The thermogram of the gene expression pattern in MSCs and MSC^SPIO^. **B** Gene Ontology (GO) enrichment analysis of all differentially expressed genes. **C**, **D** The heat map and volcano figure showed fold-changes of genes in MSCs versus MSC^SPIO^. **E**, **F** Analysis of differential expression genes using the KEGG pathway database.

Next, to further investigate the mechanism underlying the therapeutic efficacy of MSC^SPIO^, we determined the mRNA expression profile of MSCs and MSC^SPIO^ to investigate the specific effect of labeling. The whole transcriptome was sequenced by transcriptome sequencing technology (Fig. 3A). Results showed that SPIO labeling could activate many signal transduction pathways and the top 20 of GO enrichment were mainly related to lipid metabolism, inflammation, and oxidative stress (Fig. 3B). Since SPIO labeling inevitably results in iron deposition in MSC^SPIO^ and most of the changes could cause unrestricted lipid peroxidation, we speculated that SPIO labeling may induce MSCs ferroptosis.

To further verify the sequencing results, we performed a PCR array on MSCs and MSC^SPIO^ to analyze the gene expression pattern in the ferroptosis-related signaling pathway. Among genes with significant fold-change, ferroptosis suppresser *TF* [24, 25], and *CA9* [26, 27], were the most downregulated, and ferroptosis promoter *AKR1B10* [28] was the most upregulated gene (Fig. 3C, D). Active signaling pathways in MSC^SPIO^ were analyzed using the KEGG pathway database (Fig. 3E). Analysis results showed that iron metabolism changed most during ferroptosis (Fig. 3F). Our data indicate that SPIO could induce MSCs ferroptosis via iron metabolism.

**Fig. 4:**
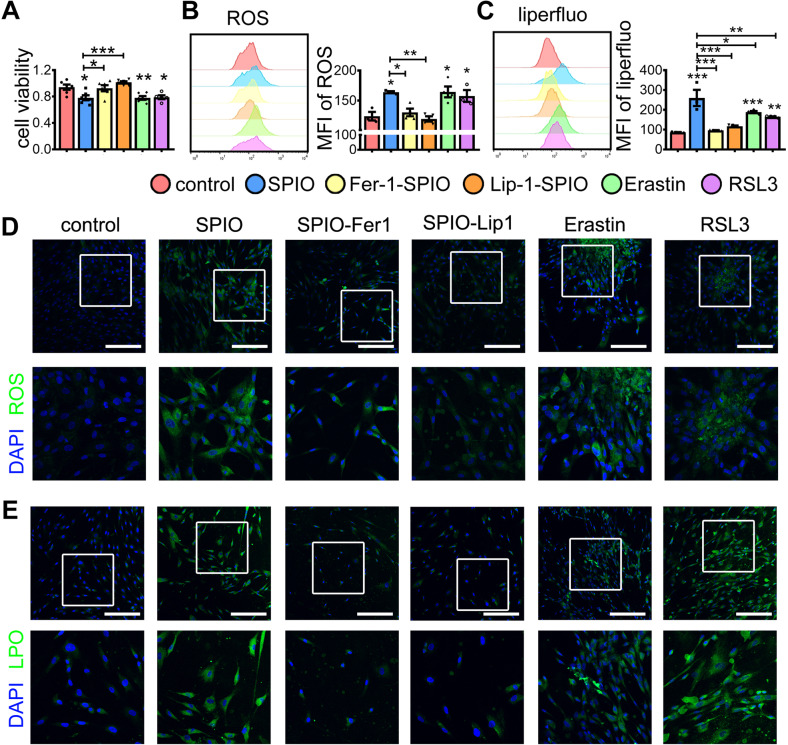
Fer-1 and Lip-1 reduced the ferroptosis of MSC^SPIO^. MSCs were treated with Erastin (100 nmol/L) or RSL3 (10 nmol/L) or treated with Fer-1 (10 μmol/L) or Lip-1 (20 μmol/L) during SPIO labeling. Cells were harvested at 24 h. **A** Cell viability of MSCs under different treatment was measured by CCK-8 assay. **B**, **D** The intracellular ROS level was measured by **B** flow cytometry and **D** confocal microscope. **C**, **E** The intracellular level of lipid peroxide was measured by **C** flow cytometry and **E** confocal microscope. Scale bars, 200 μm. Data are shown as mean ± SEM (*n* = 3). *p < 0.05; **p < 0.01; ***p < 0.001.

We next examined the level of ROS and lipid peroxide in MSCs after different treatments. The Ferroptosis inhibitor Ferrostatin-1 (Fer-1) or Liproxtatin-1 (Lip-1) treatment elevated the cell viability of MSC^SPIO^ (Fig. 4A), reduced ROS (Fig. 4B, D), and lipid peroxide level in MSC^SPIO^ (Fig. 4C, D). The Ferroptosis inducers Erastin and RSL3 were used as the positive control. Both of them reduced the cell viability of MSCs (Fig. 4A) and induced the level of ROS and lipid peroxide in MSCs (Fig. 4B–D). Moreover, SPIO downregulated the expression of GPX4 in MSC^SPIO^, while Fer-1 and Lip-1 upregulated it (Supplemental Fig. S4B). Collectively, these data confirmed that SPIO labeling promotes MSC ferroptosis.

### Ferroptosis of MSC^SPIO^ enhances the efferocytosis of macrophages

**Fig. 5:**
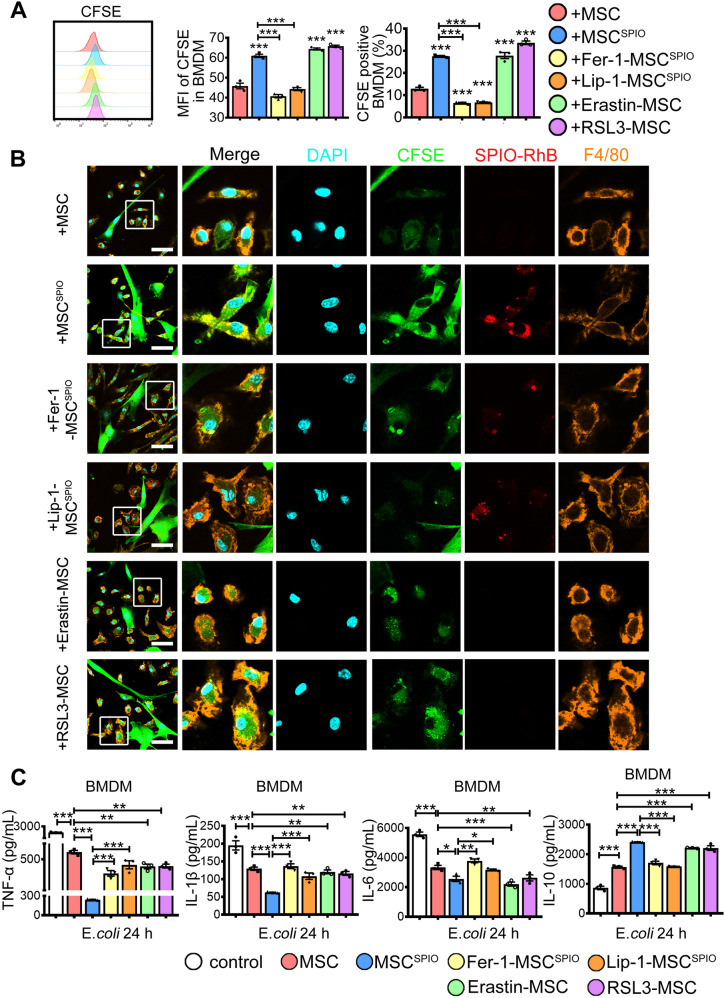
MSC^SPIO^ promotes the efferocytosis of macrophages via ferroptosis. MSCs, MSC^SPIO^, MSC^SPIO^ pretreated with Fer-1 (10 μmol/L) or Lip-1 (20 μmol/L), MSCs pretreated with Erastin (100 nmol/L) or RSL3 (10 nmol/L) were co-cultured with BMDM. **A**, **B** Phagocytosis of BMDM from each group was determined by **A** flow cytometry and **B** confocal microscope at 24 h. **C** BMDM from each group were stimulated with *E*. *coli* for 24 h, and BMDM without co-culture were used as control. Cytokines production of TNF-α, IL-1β, IL-6, and IL-10 was determined by ELISA. Data are shown as mean ± SEM (*n* = 3). *p < 0.05; **p < 0.01; ***p < 0.001.

In our previous study, we found that SPIO-treated MSCs relieved liver injury in septic mice in a macrophage-dependent manner [22]. Since macrophages play critical roles in the resolution of tissue injury via efferocytosis and MSC^SPIO^ were declined after 10 days of infusion, we next aimed to investigate the direct effect of MSC^SPIO^ on macrophages. MSCs were labeled with CFSE after different treatments and then co-cultured with bone marrow-derived macrophages (BMDM). BMDM tend to engulf more ferroptotic MSCs than Fer-1 or Lip-1-treated MSC^SPIO^ (Fig. 5A and Supplemental Fig. S5A), and SPIO in MSC^SPIO^ were transferred into macrophages during efferocytosis (Fig. 5B). Similarly, the BMDM phagocytosis enhanced when they co-cultured with Erastin or RSL3-pretreated MSCs (Fig. 5A and Supplemental Fig. S5A). Collectively, our data confirm that ferroptosis of MSCs could enhance macrophage efferocytosis.

Reports showed that dying cells can recruit macrophages to clear themselves by releasing “find-me” and “eat-me” signals [29–31], and reduce macrophages secreting inflammatory factors by TIM4 binding [32]. Apoptosis cells binding to MERTK and AXL on macrophages also inhibited the activation of Toll-like receptors and thus reduced inflammation [33]. MSC^SPIO^ significantly enhanced *TIM4* and *AXL* mRNA expressions, while it slightly increased *MERTK* mRNA expression in BMDM. Both Fer-1 and Lip-1 treatment decreased the mRNA expressions of *TIM4*, *AXL*, and *MERTK* (Supplemental Fig. S5B). When cells were stimulated with *E*. *coli* for 24 h, BMDM co-cultured with ferroptotic MSCs secreted less pro-inflammatory factors TNF-α, IL-1β, IL-6, and more anti-inflammatory factor IL-10 than the Fer-1 or Lip-1 groups (Fig. 5C). Our data indicated that SPIO labeling enhances the efferocytosis of macrophages by promoting MSC^SPIO^ ferroptosis.

### Ferroptotic MSCs protect mice against sepsis

**Fig. 6:**
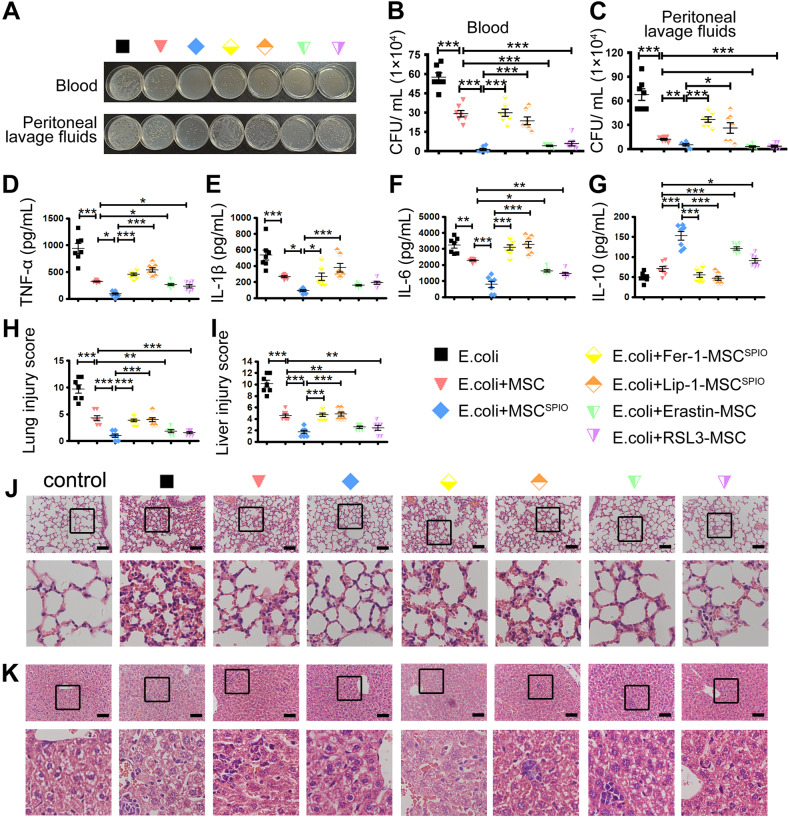
MSC^SPIO^ protect mice against sepsis via ferroptosis. Mice were injected with 2 × 10^7^ CFU *E*. *coli* intraperitoneally and then injected with 1 × 10^6^ MSCs, MSC^SPIO^, MSC^SPIO^ pretreated with Fer-1 (10 μmol/L) or Lip-1 (20 μmol/L), MSCs pretreated with Erastin (100 nmol/L) or RSL3 (10 nmol/L) intravenously. Blood and peritoneal lavage fluid from mice 4 h after infection was plated for 16 h. **A** The representative plate shows bacterial colonies. **B**, **C** Colony counting of samples from **B** blood and **C** peritoneal lavage fluid. **D**–**G** Cytokines levels in sera were determined by ELISA. **H**–**K** Mice were treated as described before and sacrificed at 48 h after infection. Representative H&E staining of **J** lung sections or **K** liver sections, and histological injury of the **H** lungs and **I** livers were scored as described in Materials and Methods. Data are shown as mean ± SEM (*n* = 7). *p < 0.05; **p < 0.01; ***p < 0.001.

To verify the therapeutic mechanism of ferroptotic MSCs on sepsis, mice were first injected intraperitoneally with *E*. *coli* 2 h and then injected intravenously with MSCs, Erastin or RSL3-pretreated MSCs, MSC^SPIO^ and Fer-1 or Lip-1-pretreated MSC^SPIO^. MSCs treatment remarkably promoted the clearance of bacteria in the blood, and the bacteria burden was the lowest in mice treated with MSC^SPIO^ (Fig. 6A–C). Treatment of ferroptotic MSCs also promoted the clearance of bacteria in the peritoneal cavity, while both Fer-1 and Lip-1 treatment reduced it (Fig. 6A–C). The pro-inflammatory factors TNF-α, IL-1β, and IL-6 levels were increased in the sera of septic mice, while all factors decreased after MSCs treatment. Moreover, the pro-inflammatory factors decreased more after treatment of ferroptotic MSCs, while the factors elevated if MSC^SPIO^ were pretreated with Fer-1 or Lip-1 (Fig. 6D–F). The level of IL-10 was increased in the sera of mice from MSCs and ferroptotic MSCs group, while it decreased with Fer-1 or Lip-1 treatment (Fig. 6G). In addition, treatment of ferroptotic MSCs decreased hyperemia and incrassation in the alveolar walls (Fig. 6H, J) and decreased sinusoidal congestion and vacuolization in the liver (Fig. 6I, K). Nevertheless, the effect of MSC^SPIO^ was impaired by Fer-1 and Lip-1 pretreatment (Fig. 6H–K). These data confirmed that MSC^SPIO^ protects mice against sepsis by ferroptosis.

Collectively, our results indicate that MSC^SPIO^ mainly aggregate to the damaged site of septic mice and induce efferocytosis of macrophages via ferroptosis to alleviate inflammation and relieve organ injury, thus inducing long-term protection against sepsis.

## Discussion

SPIO uptake by MSCs is rather difficult. Castaneda et al. found that protamine sulfate can improve the uptake of SPIO for the first time, yet the concentration of SPIO during labeling was 400 μg/mL [34]. Two years later, they reported a new method to label MSCs with SPIO in vivo, yet the concentration of SPIO was higher, and the new method was unable to label exogenous MSCs [35]. Recently, many researchers, including our team, prefer to coat SPIO with poly-lysine to improve the labeling [36]. However, this may cause enhanced cell toxicity and it requires tedious steps and extra time. The mixture of SPIO and poly-lysine had to be treated with ultrasound for 3 h, then washed and filtered to degerming. MSCs were labeled with poly-lysine coated SPIO at 200 μg/mL for 48 h. Nevertheless, we also found that the nanoparticles agglomerated sometimes when we coupled poly-lysine-coated SPIO with fluorescein. Even ultrasonication could not break up the agglomeration. Therefore, in the present study, we used the transfection agent instead.

The transfection agent was mixed with SPIO in DMEM/F-12 for 1 h, then MSCs were cultured with the mixture for 24 h, where the concentration of SPIO was 25 μg/mL. Ultrasonication, wash, and filtration were all omitted, thus shortening the time for pretreatment and labeling and saving the usage of SPIO. Agglomeration and sedimentation of nanoparticles never occurred during the treatment. More importantly, the labeling efficiency of MSCs using the transfection agent was superior to poly-lysine when compared with results in our previous study [22].

On one hand, MSCs can improve the microenvironment via the direct effect of cytokines, chemokines, extracellular vesicles, and exosomes [37]. Our previous study has shown that proper pretreatment enhanced the therapeutic effect of MSCs by regulating anti-inflammatory agents in the exosome [38]. On the other hand, MSCs exert a beneficial effect indirectly by modulating the function of neighboring immune cells [39]. Since the number of cells for injection is limited and the excess of MSCs may cause thrombosis, the engraftment rate of MSCs documented in injured areas is crucial. Therefore, various tracer products have been developed to monitor MSCs in vivo. Among them, Ferumoxytol, one of the SPIO agents, has been clinically approved by FDA as an iron supplement and is widely used for MSC tracing [40, 41]. Although Erastin and RSL3 could induce ferroptosis in MSCs in a dose-dependent manner, they are unstable in solution and unable to track the MSCs in vivo. SPIO could couple with small-molecule fluorescein for better imaging, with drugs or antibodies for better therapeutic effect [23, 42]. Besides, SPIO could be combined with heat and magnetic therapy and thus increase their clinical effect. SPIO have much more therapeutic potential than agonists currently. Moreover, we found that MSCs are more sensitive to ferroptosis than macrophages. When macrophages were stimulated with Erastin at 12.5 μM, the cell viability decreased by 10%, but the cell viability of MSCs decreased by 20% at 100 nM. This may be the reason why SPIO can cause ferroptosis in MSCs, but not in macrophages that phagocytized MSC^SPIO^.

After injection, MSCs will be dead in the face of hypoxia and inflammatory factors in vivo [43]. We also found that the cell viability was significantly decreased and the ROS level was elevated by bacteria (Supplemental Fig. S6). Since cell death is inevitable, the regulation of immune cells through efferocytosis plays an important role in MSCs therapy [44]. Mezey et al. found that nearly half of MSCs were phagocytosed by macrophages rapidly after injection [45, 46]. MSCs-derived IL-6, IDO, and TSG-6 could reprogram macrophage polarization directly. When macrophages were eliminated by clodronate liposomes, the anti-inflammatory effect of MSCs was undermined [47]. Reports showed that macrophages are important for the therapeutic effect of MSCs in kidney injury [48], myocardial ischemia/reperfusion [49], asthma [50, 51], and gut injury [52]. Our previous study also showed that macrophages are essential for the benefit of MSC therapy in sepsis [22]. In this study, we found that the enhanced efferocytosis performed by macrophages improved the therapeutic effect of MSCs, indicating that MSC therapy relies on regulating the macrophage function to achieve the desired therapeutic effects.

Early research showed that apoptotic MSCs failed to reduce inflammation in mice with *E*. *coli*-induced sepsis [53] and dead MSCs even worsened allergen-induced asthma [54]. However, recent research showed that apoptotic MSCs are still immunosuppressive and better treat GvHD [55] and they can protect mice against sepsis induced by cecal ligation and puncture (CLP) [56]. When compared with apoptotic MSCs, the migration and adaptability of living MSCs are stronger. More MSCs could be recruited to the damaged sites and respond differently according to different environments if they are alive. Although SPIO-induced ferroptosis modestly reduced the cell viability of MSCs, SPIO did not cause massive apoptosis or death in a short time, which could explain the superior effect on MCSs of ferroptosis than apoptosis or necrosis. A previous study showed that MSCs could offer an immune-privileged niche to bacteria and protect them from pro-inflammatory proteins such as TNF-α [57]. In our study, we also found that though MSCs could not take in *E*. *coli*, *E*. *coli* could invade MSCs, but SPIO labeling reduced the invasion (Supplemental Fig. S7). We speculate that ferroptosis could reduce the shielding effect of MSCs on bacteria and thus enhance their therapeutic effect.

We found that SPIO was stable in MSC^SPIO^ for 7 days in vitro, while the fluorescence of SPIO-NIR was sparkling in livers for more than 10 days. Unfortunately, the fluorescence in the liver slide was too dim to be detectable by the confocal microscope at the end of the experiment. The co-location of SPIO-NIR and MSCs remains unknown 10 days after injection. MSC^SPIO-NIR^ may have been dead and phagocytosed by macrophages, and SPIO-NIR had been transferred to macrophages at that time. Although we have monitored the spatiotemporal distribution of MSC^SPIO^ in vivo, their fate in lungs and livers is largely unknown and is extremely difficult to be monitored.

Currently, most septic mice were sacrificed at 24 h after modeling due to the distinct injury in organs, whereas we found that injury in lungs started as early as 3 h after infection and significant damage lasted for at least 3 days if mice were not treated. We also found that injury in livers started at 12 h after infection and lasted for a week, indicating that long-term damage in the livers may be one of the important causes of death in septic patients. Reports showed that MSCs injection before infection could prevent mice from sepsis [58], while we showed that MSCs injection after infection could protect mice against sepsis and alleviate tissue injury rapidly. Exploring the optimal timing of MSC therapy is key to improving septic patients’ survival rate and prognosis.

## Conclusion

In our present study, we determined the spatiotemporal distribution of MSC^SPIO^ in healthy mice and septic mice and found it can be altered by the microenvironment. MSC^SPIO^ tend to accumulate in damaged lungs and livers rapidly and enhance efferocytosis of macrophages by ferroptosis. Then the inflammatory factors secreted by macrophages were decreased and inti-inflammatory factors were increased thus MSC^SPIO^ protected mice against sepsis. Our study provided a new sight for the clinical application of MSCs and MSC^SPIO^.

## Supplementary information

Supplementary materials

Reproducibility checklist

## Data Availability

All data generated during this study are included either in this article or in the supplementary information files.
