# Supplementary material for: Ferroptotic MSCs protect mice against sepsis via promoting macrophage efferocytosis
Source: Cell Death Dis. 2022 Sep 26;13(9):825. doi: 10.1038/s41419-022-05264-z (PMC9512818; doi:10.1038/s41419-022-05264-z)
Supplement: Supplementary file 1 — Supplementary materials [file 41419_2022_5264_MOESM1_ESM.docx]

**Supplementary material**

Table S1. Antibody used for flow cytometry

| Name | Brand | Cat. No. |
| --- | --- | --- |
| APC anti-mouse F4/80 Antibody | BioLegend | 123115 |
| FITC anti-mouse/human CD11b Antibody | BioLegend | 101216 |
| FITC anti-human CD19 antibody | BioLegend | 302206 |
| PE anti-human CD73 antibody | BioLegend | 344003 |
| PE anti-human CD45 antibody | Invitrogen | 12-0459-41 |
| PE anti-human CD105 antibody | Invitrogen | 12-1057-42 |
| PE anti-human CD90 antibody | Invitrogen | 12-0909-42 |
| PE anti-human HLA-DR antibody | Invitrogen | 12-9956-41 |
| PE anti-human CD34 antibody | Invitrogen | 12-0349-41 |
| FITC mouse IgG1 kappa Isotype Control | BioLegend | 400107 |
| PE mouse IgG1 kappa Isotype Control | Invitrogen | 12-4714-41 |
| APC Rat IgG2a kappa Isotype Control | Invitrogen | 17-4321-81 |
| PE/Cyanine7 Rat IgG2a kappa Isotype Control | Invitrogen | 25-4321-82 |

Table S2. Sequences of forward and reverse primers used for PCR amplification

| Gene | Forward Primer | Reverse Primer |
| --- | --- | --- |
| β-actin | AGGTGACAGCATTGCTTCTG | GGGAGACCAAAGCCTTCATA |
| MERTK | CTCCTGAGCCCGTCAATATCT | AGACCAGGTACGGTTAGGACA |
| AXL | CTGGCTGATAACACCCAGACC | GAGCTGACTGACAACTTTCCATT |
| TIM4 | GGCTCCTTCTCACAAGAAACCACA | TCAGCTGTGAAGTGGATGGGAGA |

**Supplementary Figure**

**
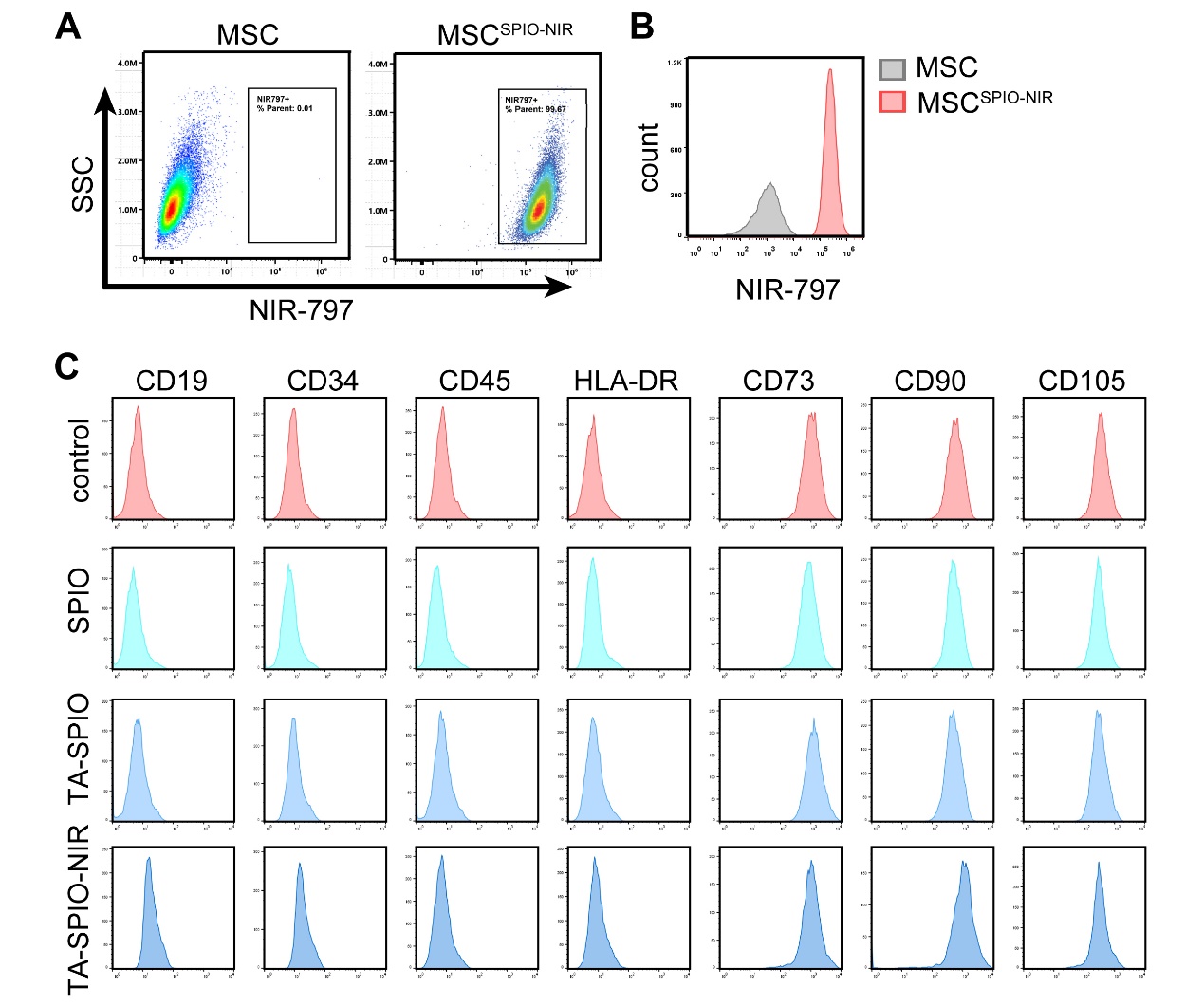
**

**Supplementary Figure S1. Characterization of MSCs and SPIO-labeled MSCs.** (A-B) MSCs were labeled with SPIO-NIR, and the fluorescence was determined by spectrum flow cytometry. (C) Cell markers of MSCs under different conditions were determined by flow cytometry.


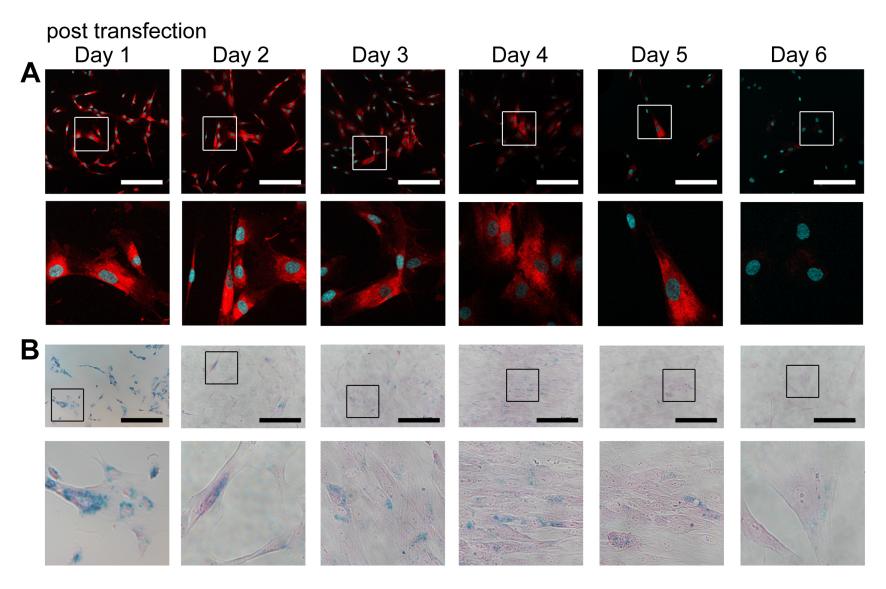


**Supplementary Figure S2. SPIO can be detectable in MSC^SPIO^ for a week.** MSCs were labeled with SPIO-NIR or SPIO as described before, and then washed 3 times and cultured with fresh culture medium for another few days. (A) SPIO-NIR in MSC^SPIO-NIR^ were measured by the confocal microscope, and (B) SPIO in MSC^SPIO^ were measured by Prussian blue staining.

**
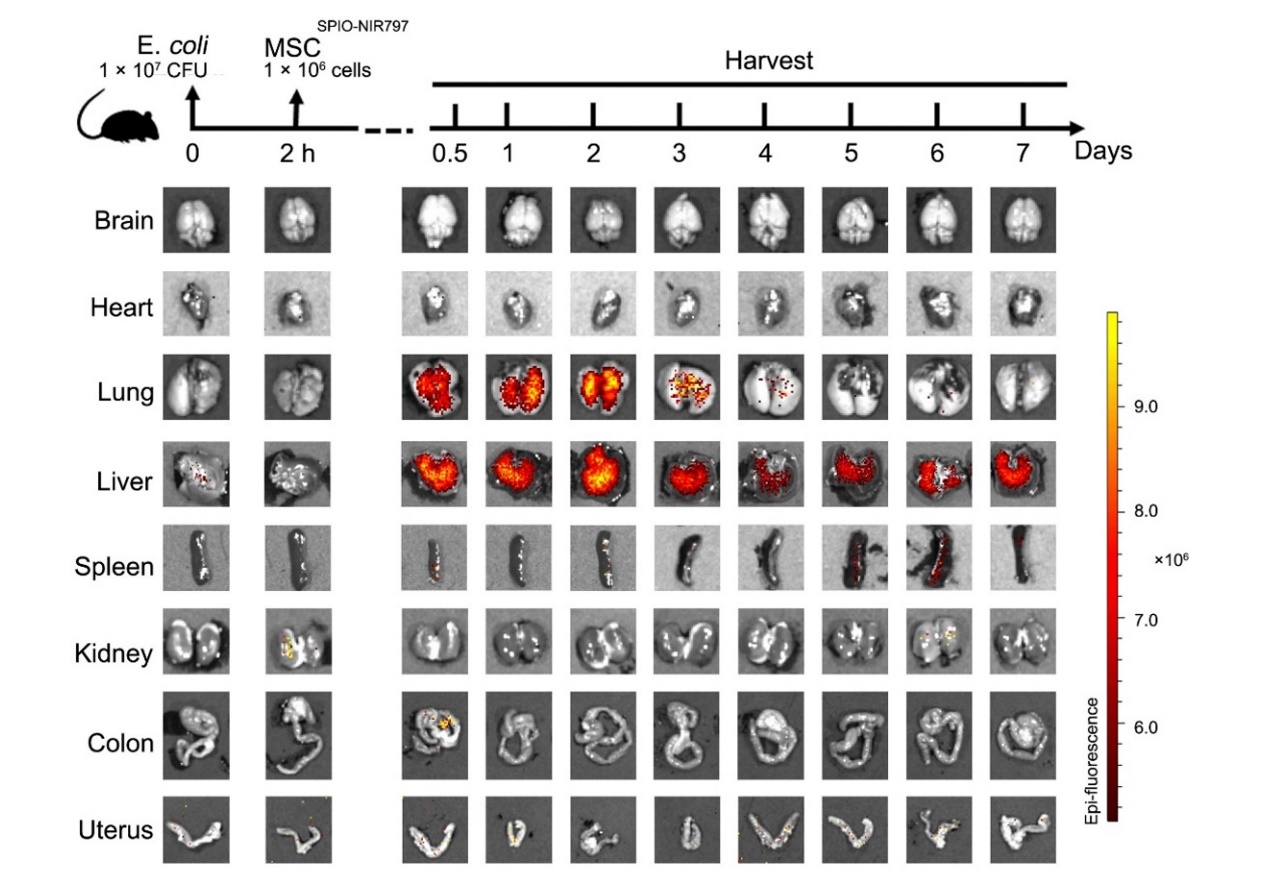
**

**Supplementary Figure S3. The spatiotemporal distribution of MSCs.** Mice were injected with MSC^SPIO-NIR^ at 2 h after E. *coli* infection and sacrificed at different time. The ex vivo imaging of major organs were carried out on the IVIS.


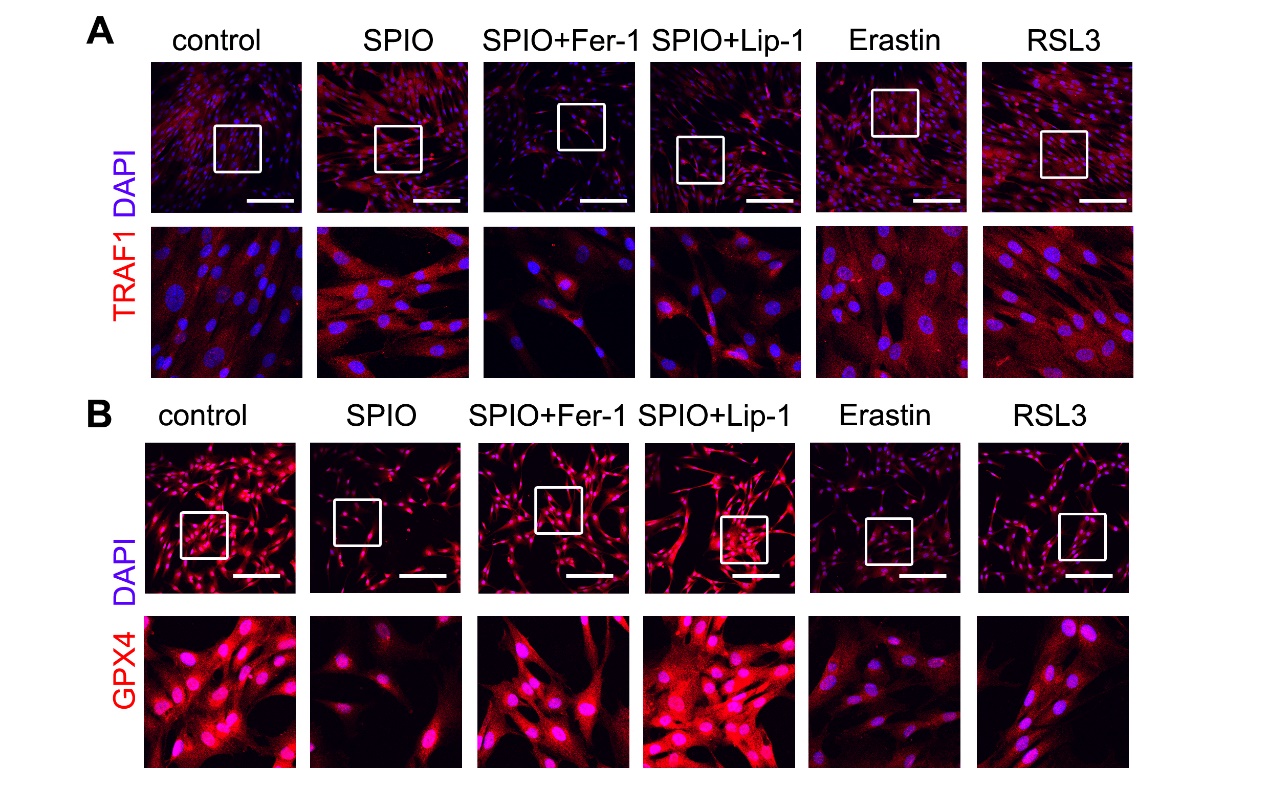


**Supplementary Figure S4. The expression of TRAF1 and GPX4 in MSCs after different treatments.** MSCs were treated with Erastin (100 nmol/L) or RSL3 (10 nmol/L) or treated with Fer-1 (10 μmol/L) or Lip-1 (20 μmol/L) during SPIO labeling. Cells were harvested at 24 h. The expressions of (A) TRAF1 and (B) GPX4 were measured by confocal microscope. Scale bars, 200 μm.

**
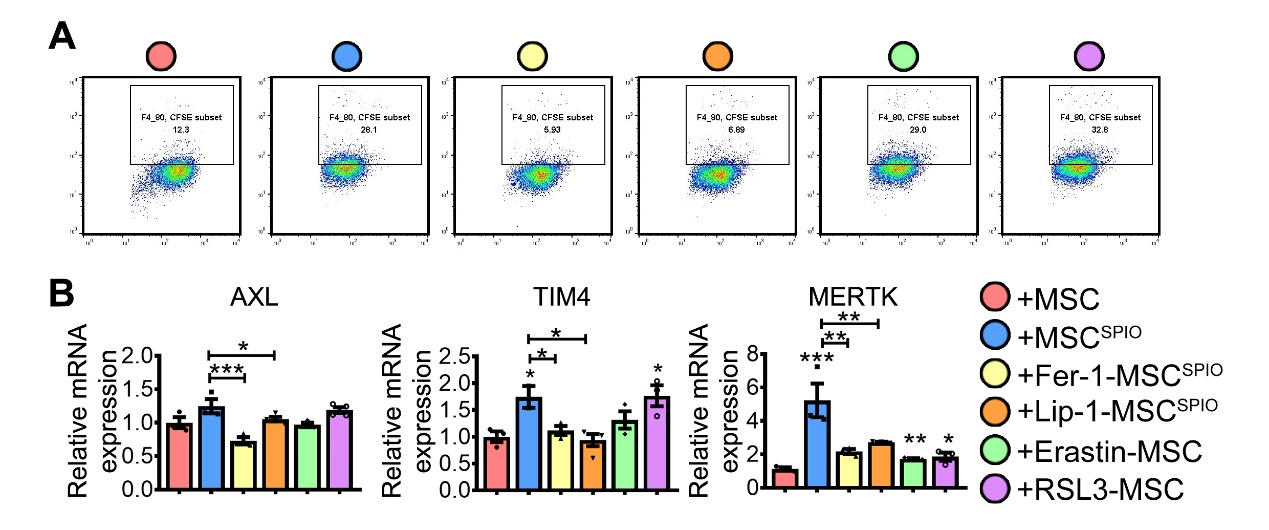
**

**Supplementary Figure S5. The efferocytosis of MSCs by macrophages under different conditions.** BMDM were co-cultured with MSCs with different treatment for 24 h. (A) The efferocytosis was determined by flow cytometry and the (B) mRNA expressions of AXL, TIM4 and MERTK were measured by quantitative PCR. Data are shown as mean ± SEM (n = 3). *P < 0.05; ***P < 0.001.


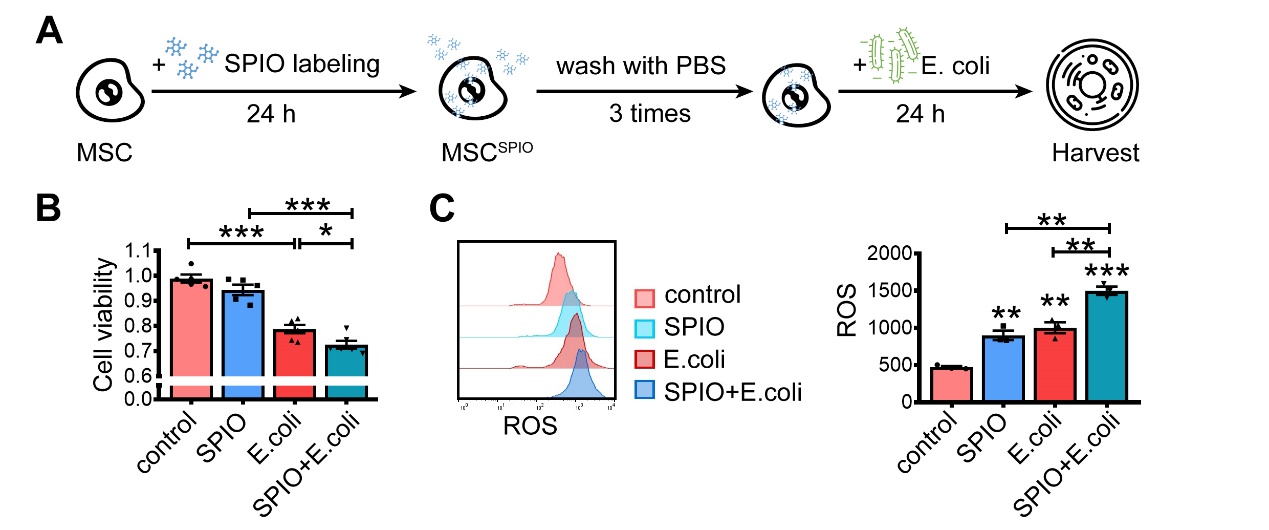


**Supplementary Figure S6. E. *coli* decreased the cell viability of MSCs.** (A) Schematic of *in vitro* experiment setup. (B) The cell viability was determined by CCK-8 assay. (B) The ROS level in MSCs was determined by flow cytometry. Data are shown as mean ± SEM (n = 3). *P < 0.05; **P < 0.01; ***P < 0.001.


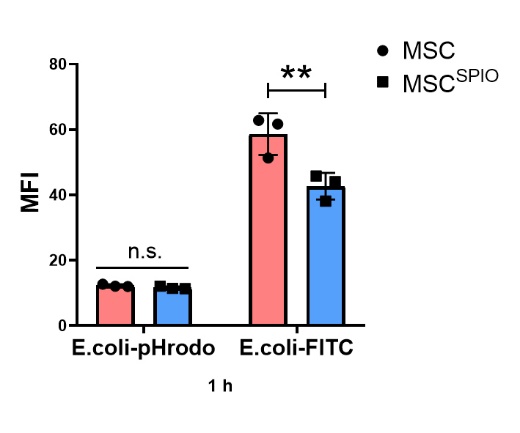


**Supplementary Figure S7. E. *coli* could invade MSCs.** MSCs or MSC^SPIO^ were co-cultured with dead E. *coli* that stained with pHrodo or E. *coli* alive that stained with FITC for 1 h. The MFI of MSCs were determined by flow cytometry. Data are shown as mean ± SEM (n = 3). **P < 0.01.
